# The regulatory mechanism of m6A modification in gastric cancer

**DOI:** 10.1007/s12672-024-00994-2

**Published:** 2024-07-15

**Authors:** Si Wu, Chunming Li, Hanghao Zhou, Ying Yang, Na Liang, Yue Fu, Qingqing Luo, YaLi Zhan

**Affiliations:** 1 Department of Pathology, The First Affiliated Hospital of Zunyi Medical University, No. 149 Dalian Street, Huichuan District, Zunyi, 563000 Guizhou China; 2 Department of Dermatology, The Second Affiliated Hospital of Zunyi Medical University, Intersection of Xinpu Street and Xinlong Street, Xinpu New District, Zunyi, 563000 Guizhou China; 3 Department of Histology and Embryology, Zunyi Medical University, No. 6 Xuefu West Street, Xinpu New District, Zunyi, Guizhou China; 4 Department of Physiology, Zunyi Medical University, No. 6 Xuefu West Street, Xinpu New District, Zunyi, Guizhou China

## Abstract

To the best of our knowledge, N6-Methyladenosine (m6A) exerts a significant role in the occurrence and development of various tumors. Gastric cancer (GC), originating from the mucosal epithelium in the digestive tract, is the fifth most common cancer and the third most common cause of cancer death around the world. Therefore, it is urgent to explore the specific mechanism of tumorigenesis of GC. As we all know, m6A modification as the most common RNA modification, is involved in the modification of mRNA and ncRNA at the post-transcriptional level, which played a regulatory role in various biological processes. As identified by numerous studies, the m6A modification are able to influence the proliferation, apoptosis, migration, and invasion of GC. What’s more, m6A modification are associated with EMT, drug resistance, and aerobic glycolysis in GC. m6A related-ncRNAs may be a valuable biomarker used by the prediction of GC diagnosis in the future. This review summarizes the role of m6A modification in the mechanism of gastric cancer, with the aim of identifying biological progress.

## Introduction

Gastric cancer (GC) is often accompanied by a low 5-year survival following initial diagnosis, which accounts for a substantial proportion of cancer-related deaths each year worldwide. As a malignant tumor, gastric cancer poses a hazard to human health all over the world. According to a 2020 report, gastric cancer, which has the fourth-highest mortality rate and the fifth-highest morbidity among 36 different types of tumors worldwide, accounts for a significant share of all malignancies in recent decades [1]. Food, lifestyle, Helicobacter pylori infection, viral infection, and other variables all have a role in what causes the development of gastric cancer. The primary trigger of gastric cancer is the stomach mucosal epithelium, which is pathologically classified mostly into intestinal types, microsatellite instability types, and mixed types. Since early gastric cancer symptoms are not typical, many individuals who are diagnosed with the disease have already entered a more advanced stage of tumor development. As a consequence, surgical resection, radiotherapy, chemotherapy, and primary chemotherapy are the main forms of treatment for gastric cancer. The pathophysiology of gastric cancer is yet unknown, nevertheless. In recent years, there has been an upsurge in the incidence and spread of stomach cancer, and research on epigenetic inheritance is becoming an increasingly prominent topic. In addition to having an aberrant expression in gastric cancer tissues, m6A regulators may interact with associated proteins to influence the activation of numerous signaling pathways, assisting in the invasion and metastasis of GC cells.

Epigenetic modifications occur in DNA, RNA and proteins, whose composition are DNA methylation, RNA methylation and histones modifications, regulating the gene expression [2]. As the most prevalent post-transcriptional modification in mammals, RNA modification is critical for the occurrence and development of tumor [3]. More than 150 different types of RNA modifications can be found in nature, which can not only occur in the no-coding RNAs (ncRNAs) involving mRNA (messenger RNA), tRNA (transfer RNA), rRNA (ribosomal RNA), snRNA (small intracellular RNA) and snoRNA (small nucleolar RNA), but also in the non-coding RNAs including microRNAs, lincRNAs and circRNAs. They are methylated in the post-transcriptional modification. It is the development of high-throughput sequencing techniques that contributing to the boom of m6A RNA modification study [4].

The methylation modification, initially found in 1974, comprised N6-methyladenosine (m6A), 5-methylcytidine (m5C) and N1-methyladenosine (m1A) modifications, which is the most widespread and prevalent chemical modifications in mRNAs and non-coding RNAs [5]. m6A modification refers to a process in which the sixth nitrogen atom on RNA is chemically modified by regulators such as “Writers”, “Erasers” and “Readers” [6]. The m6A RNA methylation modification, participating in almost all stages of RNA regulation, is involved in the transcription, processing, splicing, stability maintenance and translation of mRNA. The m6A RNA modification includes three types of molecules which are methylation recognition proteins (also called readers) that recognize the methylation binding site, methyltransferase (also known as writers), and the removal of the methylation site under the action of demethylases (also named erasers).

## The composition of m6A regulators

M6A methyltransferase, also called as “writers”, catalyzes the transfer of methyl donor from the cofactors S-adenosylmethionine (SAM) to the N6 atom of adenine. Methyltransferase complexes formed by Writers mainly include Methyltransferase-like 3 (METTL3), METTL14, METTL16, Wilms’ tumor 1-associated protein (WTAP), Vir homologous m6A methyltransferase-like (VIRMA/KIAA1429), RNA-binding motif protein 15 (RBM15/15B), zinc finger CCCH domain-containing protein 13 (ZC3H13) and Cbl proto-oncogene-like 1. METTL3, which was discovered in 1997 [7], is the most common methyltransferase. METTL3, METTL14 and WTAP form a kind of special complex named as WMM complex in the process of RNA methylation modification. What’s more important, METTL3 has a variable domain whose carbon end is mainly rich in TC bases [8]. Therefore, the main reason why METTL3 differed from METTL16 was that METTL3 has the function of transporting methyl groups. Being differed from METTL3, METTL16 methylates the 3’UTR of the SAM synthetase (MAT2A) mRNA and U6 snRNA [9]. The regulation of biological processes regarding immunity, reproduction, and the occurrence and progression of numerous diseases is greatly influenced by writers.

M6A demethylases, also known as “erasers”, stands for demethylase. Currently, the two primary demethylases are FTO and ALKBH5 (composition). Fat mass and obesity-associated protein (FTO), as a member of the non-heme Fe (II) and α-Kg-dependent dioxygenase ALKB protein family [10], whose discovery shows that methylation is a reversible process and sets the groundwork for the demethylase investigation. Alkb homolog 5 (ALKBH5), with a double-stranded B-helical core fold, is a 2-oxy-glutaric acid (2OG) and ferro-dependent nucleic acid oxygenase that catalyzes methylated adenine on RNA [11], Although FTO and ALKBH5 can reverse RNA methylation modification, their functions are different. For one thing, FTO is mainly correlated to obesity rather ALKBH5 related to fertility. For another, FTO and ALKBH5 can selectively recognize different substrates with the same consistent motifs [12]. Silencing FTO can inhibit the adipogenesis of precursor adipocytes by impeding cell cycle progression in the early stages of adipogenesis progress [13]. Demethylase is primarily responsible for removing methyl groups from target RNAs. As a result, m6A modification is a dynamic reversible process.

**Fig. 1:**
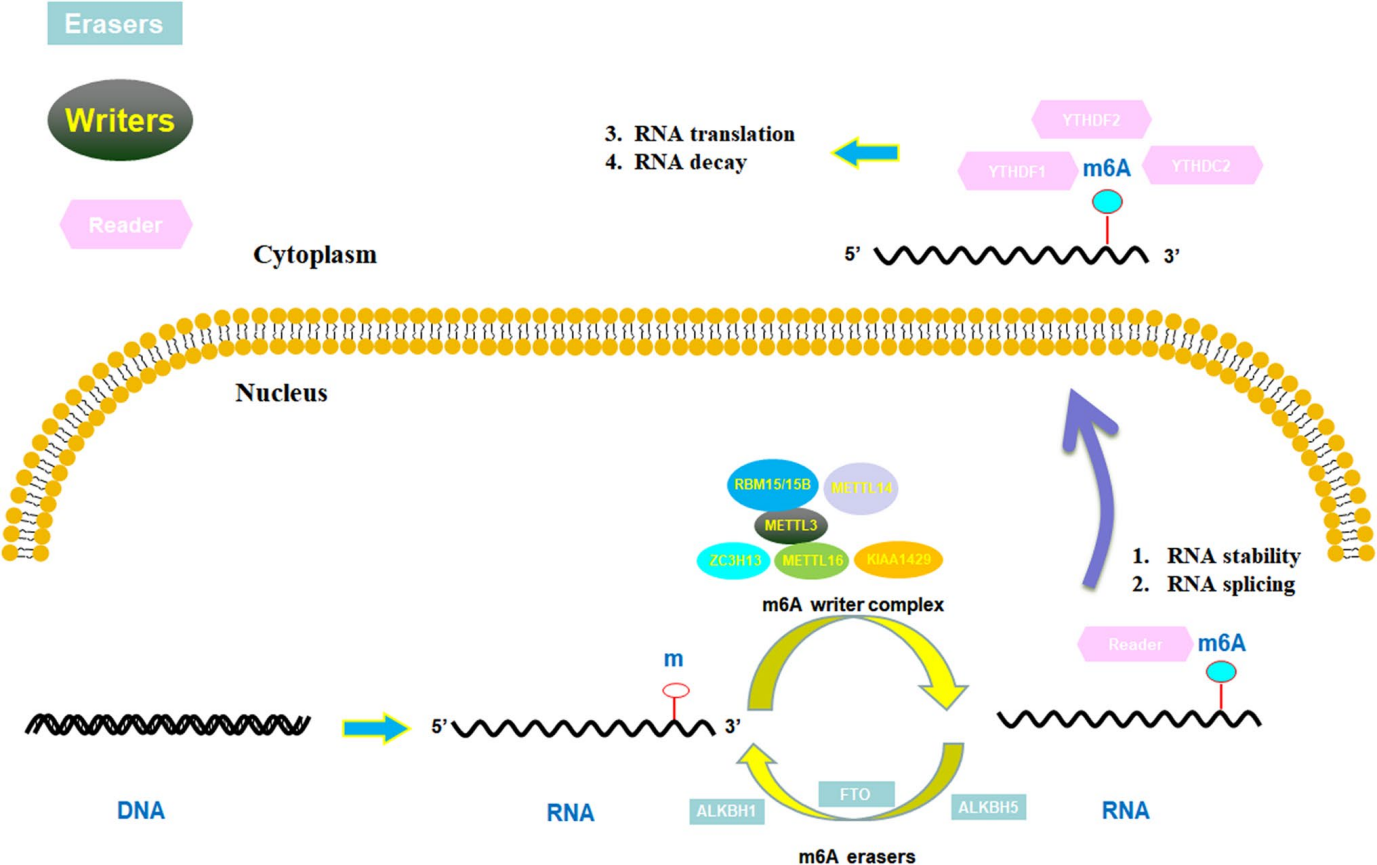
Functions of m6A regulators and the biological progress of m6A modification in GC cell. M6A modification is a reversible process. The methyltransferase consisting of METTL3 and METTL14, as well as other factors WTAP, RBM15/15B, KIAA1429, and ZC3H13(Writers) catalyzed M6A modification process. The demethylases FTO and ALKBH5 (Erasers) removed methyl groups from M6A RNA. M6A binding proteins YTHDF1-2, YTHDC2 facilitate M6A modification, which are associated with RNA stability and splicing in the nucleus and RNA translation and decay in the cytoplasm

Methylation recognition proteins, also called as “readers”, can selectively recognize and bind to targeted RNAs, participating in the expression and regulation of the downstream targeted genes. The YTH family protein, hnRNAs protein, and other m6A methylation recognition proteins make up the methylation recognition protein family at present. YTHDF2, first identified as a specific M6A recognition protein, can regulate RNA degradation of m6A methylation modification. Not only YTHDF1 and YTHDF3 can recognize methylation sites but also additionally promote the translation of targeted RNAs. As a core member of the YTH family, YTHDC1-2 is localized in the nucleus and can affect the function of RNA variable splicing and nuclear output. The functions of GC-related m6A regulators are explained in GC cell shown in Fig. 1.

## The dysregulation of m6A regulators in GC

Numerous recent studies have shown epi-transcriptomic dysregulation of m6A RNA modification in GC tissues and GC cell lines, with most showing increased m6A in comparison to that in paired adjacent normal tissues. However, the role of m6Ain the tumorigenesis and development of GC is unknown. Many studies have proved that the abnormal expression of m6A regulated factors is correlated to the biological phenotypes of GC, which is beneficial to better understand the mechanism of m6A in the carcinogenesis, development and metastasis of GC.

**Fig. 2:**
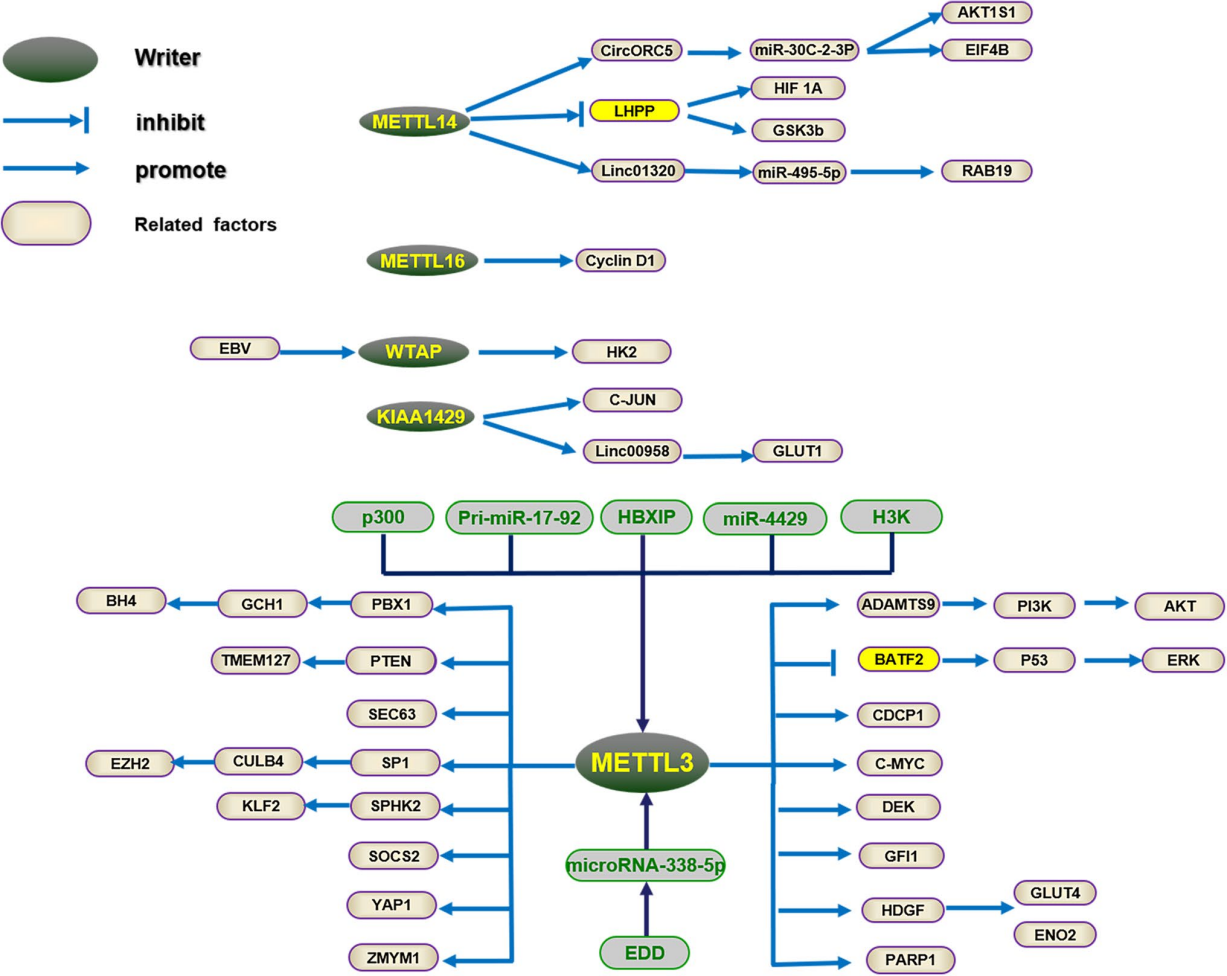
Aberrant m6A writers promote or inhibit GC by interacting with GC-related RNAs and regulating the corresponding pathways

Writers are widely studied in the m6A regulators of GC. First of all, it has been demonstrated that the METTL3, as a methyltransferase of m6A RNA modification, plays a crucial role in GC [14]. The methyltransferase complex is made up of three crucial parts: METTL14, METTL3, and WTAP. Zhang et al. [15] found that GC patients with high METTL3 expression had a poor prognosis and m6A RNA modification level of mRNA was higher in GC tissues and GC cell lines. In accordance with the clinical GC tissues, Liu et al. [16] detected that METTL3 expression was significantly increased in GC cell line and tumor tissues from The Cancer Genome Atlas (TCGA), Kaplan–Meier plotter, and Gene Expression Omnibus (GEO) database compared with control in big crowd data sets, and was related with more advanced tumor stage and grade in the survival analysis. Not only can METTL3 serve as an oncogene in GC, but also as a transcription factor such as HBXIP mRNA to participate in the METTL3 transcription process [17]. Consisted with other m6A writer complex, the expression of WTAP and RBM15 were upregulated in the GC in the recent studies. Variously, WTAP with highly expression implied a worse prognosis and served as an independent predictor indicating the low survival rate [18]. Based on a univariate cox regression analysis in the protein expression level, Survival analysis indicated that patients with gastric cancer exhibiting high RBM15 expression had a better prognosis [19]. By contrast, METTL14 is low expressed in GC cells and tissues and is correlated with oncogenic signaling and phenotypes, for the reason that METTL14 can suppress the progression of GC by deactivating the WNT/PI3K-Akt pathway and metastasis progress through regulating m6A RNA modification [20]. Through bioinformatics and immunohistochemistry analysis, Liu et al. demonstrated that METTL14 could be a potential tumor suppressor in GC by regulating the PI3K/AKT/mTOR signaling pathway and epithelial mesenchymal transition (EMT) pathway. Additionally, METTL14 is the main regulator for the abnormal m6A RNA modification, regulating the migration and invasion of GC cells via the EMT process in vitro experiments [21]. METTL14 with ectopic expression markedly inhibited the growth and invasion of GC cells in vitro and in vivo function experiments. As identified by rescue experiments and validated by methylated RNA immunoprecipitation (Me-RIP), METTL14 knockdown reduced circORC5 m6A levels, which correlated negatively with miR-30c-2-3p and indicated poor survival in GC [22]. Through GSEA enrichment analysis, the writer METTL5 was also verified to be significantly downregulated in gastric cancer tissues (GCTs) compared with adjacent normal tissues (ANTs) and adjacent intestinal metaplasia tissues (AIMTs) in a study of 168 GC patients, which are negatively associated with clinicopathologic stage and a poor prognosis [23]. Last but not least, the mechanism mediated by METTL5 in GC remains to be illustrated in the future. The functions of GC-related m6A writers, as well as their target RNAs, are explained in detail shown in Fig. 2.

**Fig. 3:**
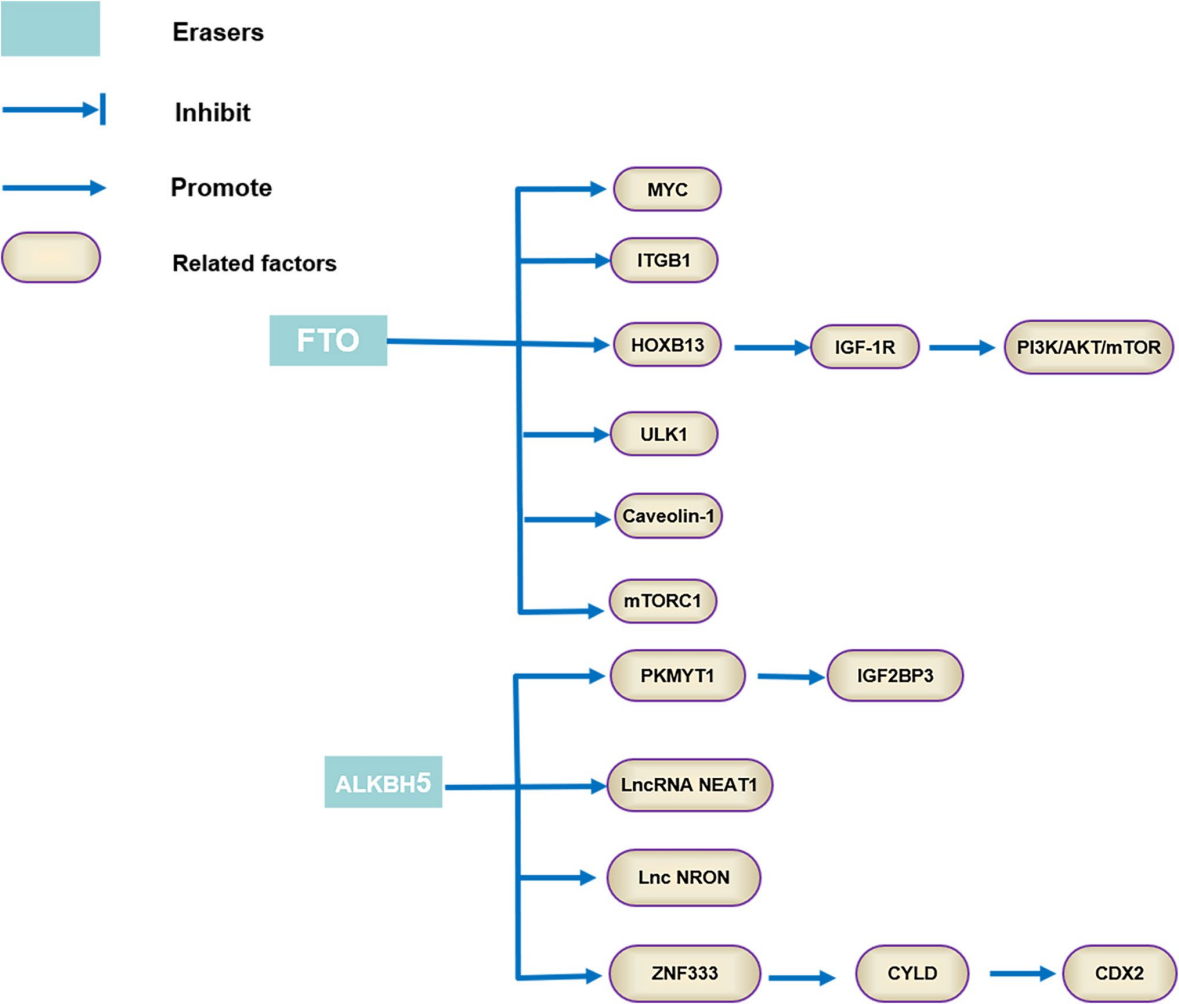
Aberrant m6A erasers promote or inhibit GC by interacting with GC-related RNAs and regulating the corresponding pathways

M6A “eraser” related gene including FTO and ALKBH5 were found to be a reliable prognostic and predictive tool in a recent study of 738 gastric cancer (GC) samples obtained from two independent datasets. Additionally, these genes were found to significantly impact the tumor immune microenvironment by influencing the expression of genes related to immune cell function and signaling pathways [24]. According to research conducted on a group of 100 gastric cancer patients, the m6A levels in the peripheral blood RNA were found to be significantly higher in comparison to those observed in both the benign gastric disease group (BGD) and healthy control group (HCs). Additionally, the study found that the expressions of two m6A demethylases, ALKBH5 and FTO, were substantially suppressed in both in vitro and in vivo samples, suggesting a possible correlation between elevated m6A levels and the development of gastric cancer [25]. High FTO expression exhibited markedly the worse overall survival (OS) and recurrence free survival (RFS) which demonstrated a poor prognosis [26]. Zhou et al. [27] reported that high FTO and ALKBH1 levels has a statistically significant association with a worse overall survival of patients with gastric cancer. However, FTO and ALKBH1 were significantly downregulated at both mRNA and protein levels in GC tissues, which were closely associated with the larger tumor sizes (≥ 5 cm) and more advanced TNM stages and shorter overall survival of GC patients [28]. The technique of Methylated RNA immunoprecipitation sequencing (MeRIP-seq) was utilized to identify changes in gene expression within GC tissues and cell lines, the expression of ALKBH5 was decreased in GC tissues and cell lines. Additionally, ALKBH5 with low expression was correlated with clinical tumor distal metastasis and lymph node metastasis in GC patients [29]. Wang et al. [30] discovered that ALKBH5 was decreased in GC samples, which was associated with the worse prognosis and was positively correlated with tumor size, tumor stage, distant metastasis, and tumor-node-metastasis (TNM) stage. However, Yue et al. [31] reported that ALKBH5 was overexpression in intestinal metaplasia (IM) tissues compared with adjacent normal gastric tissues, which may be a potential preventive and therapeutic strategy for gastric IM in GC patients. Identifying the specific demethylases that are overexpressed or silenced in certain tumors can provide valuable insights into the underlying molecular mechanisms driving tumor initiation and progression. These findings suggest that ALKBH5 may play a critical role in the development and progression of GC and could potentially serve as a useful biomarker for predicting tumor metastasis in GC patients. Further investigation into the mechanism underlying the role of ALKBH5 in GC development could shed light on new therapeutic strategies for the disease. Therefore, it is necessary that elucidate clearly the various expression of m6A regulators in GC. The functions of GC-related m6A erasers, as well as their target RNAs, are explained in detail shown in Fig. 3.

**Fig. 4:**
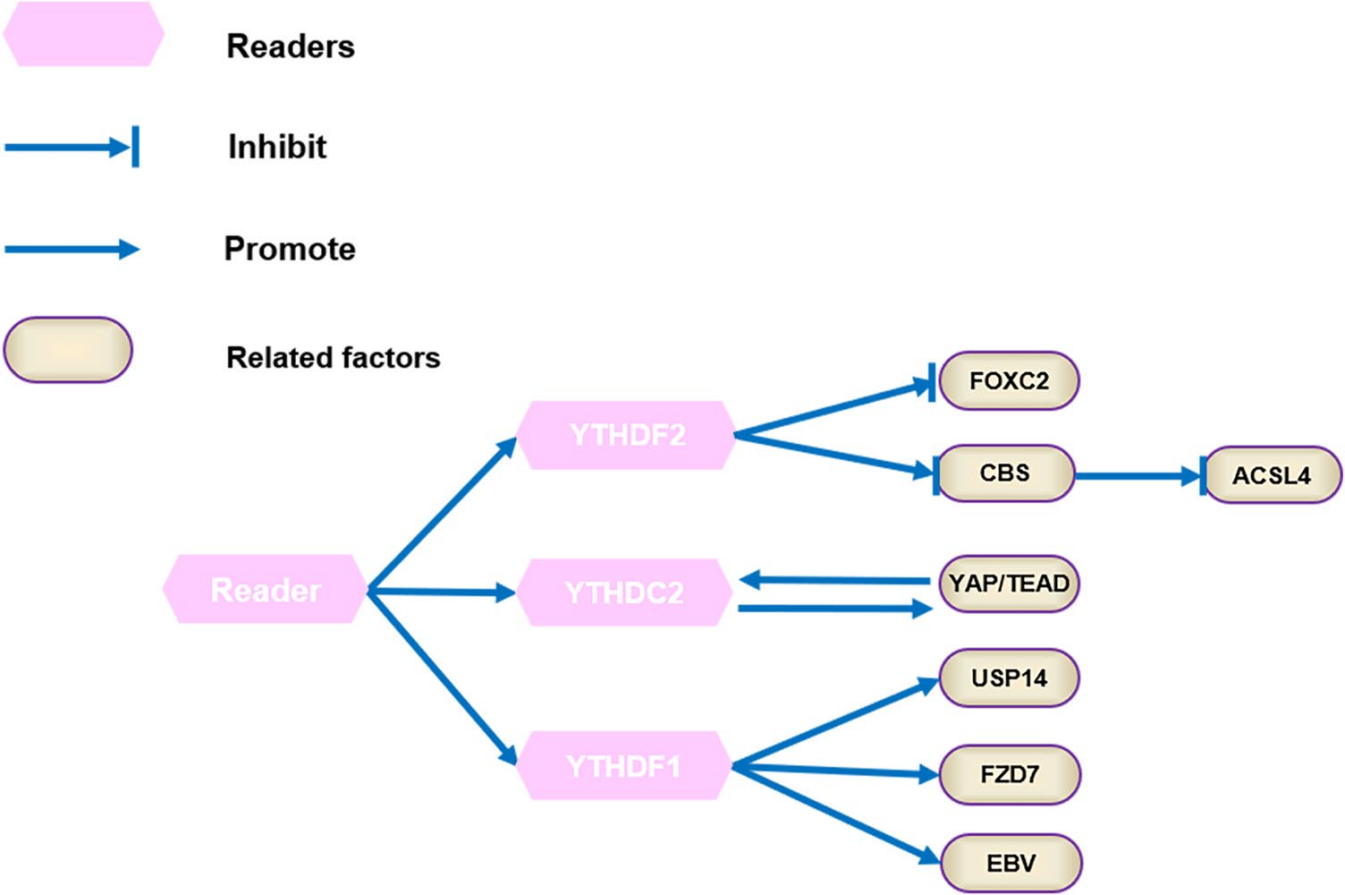
Aberrant m6A readers promote or inhibit GC by interacting with GC-related RNAs and regulating the corresponding pathways

Readers are also abnormal expression in GC samples, contributing to biological changes in m6A modification. Validated by IHC staining analysis from GC tissue microarray (TMA) cohort and Human Protein Atlas (HPA) database, YTHDF1/2/3 and hnRNPA2B1 of m6A “readers” were overexpressed at protein and gene expression level in GC, while HNRNPA2B1 and YTHDC1 had no difference in the expression level in GC [32]. Pi et al. [33] analyzed that cBioPortal (cBio Cancer Genomics Portal) datasets including 630 primary gastric adenocarcinoma patients and revealed that YTHDF1, as a general oncogenic role, was mutated in 7% of patients with gastric cancer and YTHDF1 with high expression was correlated with more aggressive tumor progression and poor overall survival. What’ more important, according to a study based on public datasets from GEO, YTHDF2 was at a low expression level in GC tissues compared to normal gastric tissue, which plays a crucial role in the clinical stage in GC [34]. Liu et al. [35] reported that YTHDF1 was upregulated in 17 pairs of GC specimens and paired adjacent non-tumor tissues and developed the m6A gene-based diagnostic signature, which was significantly correlated with the high-risk subtype of GC patients. However, it’s hard to define the clinical significance of m6A readers as biomarkers for that the different readers have various biological functions and behaviors in GC revealed in numerous studies. The functions of GC-related m6A readers, as well as their target RNAs, are explained in detail shown in Fig. 4.

As referred above, with the development of diseases, there are a multiply of dysregulation of m6A RNA modification in GC, which will contribute to various prognosis and clinical features. Gastric cancer is a complex process regulated by various m6A regulators. As a consequence, it is hard to elucidate that the specific mechanism of gastric cancer tumorigenesis in m6A RNA modification level. However, when it comes to the abnormal expression of M6A regulators such as Writes, Erasers and Readers are upregulated or downregulated in the transcriptional level in GC. What’s more, these m6A regulators compose a complex network affecting each other. To better understand the oncological mechanism of GC about m6A dysregulation, it is crucial to concentrate on downstream pathways and molecules regulated by abnormal expressed m6A regulators.

## Biological functions of m6A modification in GC

1. mRNA

The epigenetic modification was considered as a crucial part in the tumorigenesis, progression and metastasis of GC. The role of m6A RNA modification in the regulation of gene expression depends on the mRNA, the sites of m6A RNA modification, and the related m6A regulators. A multitude of studies have revealed that the methylation of m6A modification mRNA occupy an important position in the tumorigenesis and progression of tumors recently [2]. Identified by GO, KEGG enrichment analysis, m6A in both GC and PCa tissues was significantly enriched at the coding sequence (CDS) and 3’UTRs, which may be correlated with RNA stability, transport, and translocation signals or protein synthesis [36]. Chen et al. [37] identified that USP14 was a downstream target of YTHDF1, which could promote USP14 protein translation in a m6A-dependent manner. Myelocytomatosis viral oncogene homolog (MYC) presented increased mRNA expression compared to the paired control samples in GC, which associated with deeper tumor extension (p = 0.006), lymph node metastasis (p = 0.023), and distant metastasis (p < 0.001) [38]. MYC, as an oncogene, are related to initiation and progression of GC. Yang et al. [17] detected the MYC mRNA in transcription level through m6A RNA immunoprecipitation and dot-blot assays, and elucidated that METTL3 can manipulate the methylation of MYC mRNA, which facilitates the translation of the MYC protein by increasing its stability and subsequently promotes the GC progression. A recent study revealed that not only IGF2BP1 can promote the proliferation, metastasis and prognosis of GC, but also facilitate its targeted mRNA c-MYC to accelerate glycolysis (also known as Warburg effect) in a m6A/c-MYC-dependent manner [39]. Integrated RNA-seq and m6A-seq analysis firstly indicated that several component molecules of MYC target genes (such as MCM5, MCM6,) were mediated by METTL3 through altered m6A RNA modification. There is a decrease in the stability of MCM5 and MCM6 transcripts [40]. To the best of our knowledge, not only m6A-modified mRNAs are dysregulation, but also participate in the biological process in GC. The methylation of HDGF mRNA mediated by METTL3 enhances mRNA stability and was directly recognized by the m6A reader IGF2BP3 that bound to the m6A site of HDGF mRNA, which facilitates subsequent tumor growth and liver metastasis in GC [41]. On the contrary, after knocking out expression of METTL3, the activity of the YAP1 signaling pathway and the proliferation and metastasis of gastric cancer were inhibited by modifying the m6A RNA modification level of the YAP1 gene [42]. More importantly, Knock-down METTL16 decreased the overall level of m6A and the stability of cyclin D1 mRNA which means the inhibition of methyltransferase activity in GC cells. Meanwhile, the cell cycle was blocked in G1/S phase and the cyclin D1 expression was significantly reduced at both mRNA and protein levels, which are associated with the proliferation of GC cells [43]. LHPP, regulated by m6A methylation and acting as a tumor suppressor in GC, influenced cell proliferation, invasion, and drug resistance and regulated the metabolism of GC by changing the acetylation level. Lin et al. [44] conducted an enrichment analysis of the reactome pathway and the biological functions of LHPP, revealing that LHPP is significantly related to the Akt signaling pathway, WNT signaling pathway, and cell energy metabolism pathways. YTHDF1 expression could inhibit gastric tumorigenesis by altering the translation of a key WNT receptor frizzled 7 (FZD7) in an m6A-dependent manner and regulating WNT/β-catenin signaling in gastric cancer [33]. Low expression of YTHDF1 prolonged the half‐lives of the BRLF1 mRNAs and lead to lytic replication and progeny production of Epstein–Barr virus (EBV), which promotes EBV infection and replication. Conversely, YTHDF1 promotes viral RNA decay [45]. With m6A suppression (represented for METTL14 knockdown) in GC, WNT and PI3K-Akt signaling was activated, promoting the proliferation and invasion of GC cells, while m6A elevation (represented for FTO knockdown) reversed these phenotypical and molecular changes [20]. ITGB1, a demethylated target whose m6A RNA modification sites are generally located in the 3′-UTR of mRNA of FTO in GC, played a role in the growth of multiple tumors. The increased ITGB1 expression promoted migration and invasion abilities of GC cells via decreasing its mRNA m6A RNA modification level [46]. Furthermore, one recent study revealed a special function of mRNA m6A RNA modification in GC. KIAA1429, serving as a scaffold that bridges the catalytic core components of the m6A methyltransferase complex, acted as an oncogene in gastric cancer by stabilizing c-Jun mRNA in an m6A-independent manner [47]. Remarkably, YTHDC2 recognized m6A-modified YAP mRNA at 5`-UTR region, which enhanced the translation efficiency of YAP instead of affecting YAP mRNA level [48]. As mentioned above, the mechanisms regulated by m6A RNA modification are intricated in GC-related mRNAs that played a crucial role in the tumorigenesis and progression of GC. The mechanisms by which GC-related mRNAs are regulated by m6A modification are summarized in Table 1.

(2) ncRNA

**Table 1:** m6A-regulated mRNAs in gastric cancer

mRNA	m6A regulators		Pathway	phenotype	Role of target	Function of regulator	Reference
ZMYM1	METTL3	Writer	METTL3/ZMYM1/E-cadherin	EMT program and metastasis	METTL3 promoted m6A by enhancing ZMYM1 mRNA stability	Oncogene	METTL3-mediated m6A modification is critical for epithelial-mesenchymal transition and metastasis of gastric cancer
	HuR	Reader					
HDGF	METTL3	Writer	MTTL3/HDGF	Glycolysis, proliferation and liver metastasis	Promoting m6A and inducing METTL3 transcription by enhancing HDGF mRNA stability	Oncogene	METTL3-mediated m6A modification of HDGF mRNA promotes gastric cancer progression and has prognostic significance
	IGF2BP3	Reader					
C-MYC	METTL3	Writer	METTL3/MYC	Proliferation, migration and invasion	METTL3-mediated MYC mRNA m6A modification	Oncogene	HBXIP promotes gastric cancer via METTL3-mediated MYC mRNA m6A modification
MYC	METTL3	Writer	METTL3MYC	Proliferation and migration and invasion		Oncogene	METTL3 Promotes the Progression of Gastric Cancer via Targeting the MYC Pathway
SPHK2	METTL3	Writer	METTL3/SPHK2/KLF2	Proliferation, migration and invasion	METTL3 promotes translation of SPHK2 mRNA	Oncogene	METTL3-mediated m6A methylation of SPHK2 promotes gastric cancer progression by targeting KLF2
YAP1	METTL3	Writer	YAP1/TEAD	Proliferation and metastasis	METTL3 reduces m6A methylation and the total mRNA level of YAP1	Oncogene	m6A Methyltransferase 3 Promotes the Proliferation and Migration of Gastric Cancer Cells through the m6A Modification of YAP1
DEK	METTL3	Writer	MTTL3/DEK	Proliferation and migration	METTL3 promoted m6A modification level and enrich DEK mRNA by promoting the stability of DEK mRNA	Oncogene	The m6A Methyltransferase METTL3-Mediated N6-Methyladenosine Modification of DEK mRNA to Promote Gastric Cancer Cell Growth and Metastasis
LHPP	METTL14	Writer	METTL14/LHPP	Glycolysis, proliferation, invasion and metastasis	METTL14 represses LHPP mRNA expression, regulating the metabolism of GC by changing the acetylation level	Suppressor	m6A methylation mediates LHPP acetylation as a tumor aerobic glycolysis suppressor to improve the prognosis of gastric cancer
cyclin D1	METTL16	Writer	METTL6/cyclin D1	Proliferation	METTL16 increased m6A level by enhancing cyclin D1 mRNA stability	Oncogene	METTL16 promotes cell proliferation by up-regulating cyclin D1 expression in gastric cancer
c-JUN	KIAA1429	Writer	KIAA1429/c-Jun	Proliferation	KIAA1429 could stabilize c-Jun mRNA in an m6A-independent manner	Oncogene	KIAA1429 regulates cell proliferation by targeting c-Jun messenger RNA directly in gastric cancer
MYC	FTO	Eraser	FOXA2/FTO/m6A/MYC	Cell viability, migration and invasion	FTO stabilized MYC mRNA by reducing m6A methylation of MYC	Oncogene	HDAC3-dependent transcriptional repression of FOXA2 regulates FTO/m6A/MYC signaling to contribute to the development of gastric cancer
ITGB1	FTO	Eraser	FTO/ITGB1	Migration and invasion	FTO inhibited m6A level by upregulating the expression of Integrin β1(ITGB1)	Oncogene	m6A RNA Demethylase FTO Promotes Gastric Cancer Metastasis by Down-Regulating the m6A Methylation of ITGB1
PKMYT1	ALKBH5	Eraser	ALKBH5/PKMYT1/IGF2BP3	Invasion and migration	ALKBH5 demethylase inhibited m6A level by stabilizing the PKMYT1 mRNA stability	Suppressor	Demethylase ALKBH5 suppresses invasion of gastric cancer via PKMYT1 m6A modification
	IGF2BP3	Reader					
EBV	YTHDF1	Reader	YTHDF1/EBV	EBV infection and replication	YTHDF1 hastens viral RNA decay by recruiting RNA degradation complexes	Suppressor	m6A -binding protein YTHDF1 suppresses EBV replication and promotes EBV RNA decay
FZD7	YTHDF1	Reader	WNT/β-catenin	Proliferation and tumorigenesis	YTHGDF1 control translation of key oncogenic drivers by manipulating epigenetic regulators	Oncogene	YTHDF1 Promotes Gastric Carcinogenesis by Controlling Translation of FZD7
USP14	YTHDF1	Reader	YTHDF1/USP14	Proliferation, invasion, metastasis and apoptosis	YTHDF1 promoted USP14 protein translation in an m6A-dependent manner	Oncogene	The m6A Reader YTHDF1 Facilitates the Tumorigenesis and Metastasis of Gastric Cancer via USP14 Translation in an m6A -Dependent Manner
FOXC2	YTHDF2	Reader	YTHDF2/FOXC2	Proliferation, invasion, and migration	YTHDF2 inhibits the growth of GC cells	Suppressor	YTHDF2 Inhibits Gastric Cancer Cell Growth by Regulating FOXC2 Signaling Pathway
YAPA	YTHDC2	Reader	YTHDC2/YAP	Cell viability, proliferation and invasion	YTHDC2 recognized m6A-modified YAP mRNA at 5`-UTR, resulting in enhancing the translation efficiency of YAP	Oncogene	The m6A reader protein YTHDC2 promotes gastric cancer progression via enhancing YAP mRNA translation
SIRT1	IGF2BP2	Reader	IGF2BP2/SIRT1	Proliferation and migration	IGF2BP2 regulated GC progression through recognizing the m6A modification sites of SIRT1 mRNA	Oncogene	m6A reader IGF2BP2 promotes gastric cancer progression via targeting SIRT1
C-MYC	IGF2BP1	Reader	IGF2BP1/m6A/c-MYC	Migration and aerobic glycolysis	IGF2BP1 interacted with c-MYC mRNA via m6A-dependent manner to by stabilize c-MYC mRNA stability	Oncogene	m6A reader IGF2BP1 accelerates gastric cancer aerobic glycolysis in c-MYC-dependent manner

By using high-throughput sequencing and TCGA datasets analysis, non-coding RNAs consist of the majority (about 85%) of transcriptome [49]. LncRNAs, as oncogenes or tumor suppressors, played a critical role in the development and progression of tumor, which regulated gene expression at chromosomal, transcriptional, and post-transcriptional levels [50]. Studies reported that RNA modification played a regulatory role in the post-transcriptional protein modifications through various molecular mechanisms [51]. Through lncRNA microarray analysis, Liu et al. identified THAP7-AS1 was significantly upregulated in GC tissues compared with non-tumorous gastric tissues among 1414 differentially expressed lncRNAs. LncRNA, transcriptionally activated by a transcription factor SP1 and then modified by METTL3-mediated m6A regulator, improved the CUL4B protein entry into the nucleus to repress the transcription of miR-22-3p and miR-320a [52]. Consistent with the data from TCGA, LncNRON9, as an oncogene, was high expression in GC and could bind to the N6-methyladenosine eraser ALKHB5 and regulate Nanog mRNA decay and protein levels at the post-transcriptional level [30]. Luciferase reporter assay and RNA immunoprecipitation assay experiments verified that LINC01320 is methylated by METTL3. LINC02253 expression was closely correlated with tumor size, lymph node metastasis and TNM stage of GC, which promoting GC cell growth, migration and invasion both in vitro and in vivo by activating KRT18/MAPK/ERK pathway [53]. lncRNA NEAT1 (nuclear paraspeckle assembly transcript 1), acting as a scaffold mediated by ALKBH5, binds with EZH2 to promote the expression of downstream genes of EZH2 (a subunit of the polycomb repressive complex), which affects the malignant phenotype of GC such as invasion and metastasis progression [54]. Using univariate Cox regression, LASSO regression, and multivariate Cox regression analyses, Hu et al. established a novel and potential prognostic m6A-related lncRNAs prognostic signature and reported that higher level of m6A RNA modification and lncRNAs screened in human gastric cancer were associated with the poor OS [55]. MicroRNA can also have an impact on the biological functions in GC, in addition to lncRNA, which can play a regulatory role in the carcinogenesis and progression of GC. Dual-luciferase reporter gene detection and RNA pull-down assay further confirmed LINC01320 could bind with miR-495-5p. Meanwhile, miR-495-5p can be effectively enriched in LINC01320 and be sponged with LINC01320. Therefore, miR-495-5p was predicted and demonstrated as a target of LINC01320, regulating cell viability, migration, and invasion of gastric cancer through the miR-495-5p/RAB19 axis [56]. He et al. discovered that miR-4429 inhibit m6A-caused stabilization of SEC62 mRNA and suppress GC proliferation but enhanced apoptosis through regulating METTL3 and SEC62 expression. What’ more, rescue assays demonstrated that miR-4429 inhibited GC progression via METTL3/SEC62 axis [57]. What can be inferred is that miR-4429 may serve as a promising target for treatment improvement of GC. METTL3 mediated the process of pri-miR-17-92 into the oncogenic miR-17-92 cluster through an m6A/DGCR8-dependent way, which are correlated with the proliferation and migration in vitro that consisted with the result of a vivo experiment [58]. As mentioned above, abnormal m6A RNA modification is a crucial epigenetic feature and a potential therapeutic target in gastric cancer. When it comes to the circRNAs mediated by m6A RNA modification, the mechanisms and functions of proteins mediated by m6A-modified circRNAs are little researched in GC. Fan et al. analyzed that differentially expressed circRNAs served as a poor prognostic factor in patients with GC and found that most of differentially expressed circRNAs with m6A RNA modifications were consistent with the expression levels of circRNAs. Overexpression of METTL14 markedly repressed the growth and invasion of GC in vitro and in vivo, whereas knockdown of METTL14 exerted the opposite effects [22]. Down-regulation of circPVRL was negatively associated with TNM stage (P < 0.05) and significantly shorter survival times, which exerting carcinogenesis roles by sponging miRNAs. More than that, circPVRL3 has a potential ability to encode proteins as it is abundant in the cytoplasm and may exert effects through miRNA-mediated gene expression in GC [59]. CircRNAs could regulate gene expression at the post-transcriptional level and play an important role in regulating various pathological processes in GC. Zhang et al. reported that m6A RNA modification may be prevalent in human gastric tissues and may be closely related to circRNAs in function, which provides new insights into the regulation of circRNAs in a m6A-dependent manner in PDGA and GC [60]. The mechanisms by which GC-related mRNAs are regulated by m6A modification are summarized in Table 2.

**Table 2:** m6A-regulated ncRNAs in gastric cancer

ncRNA	m6A regulators		Pathway	Phenotype	Role of target	Function of regulator	Reference
lncRNA THAP7-AS1	METTL3	Writer	METTL3/PI3K/AKT	Growth, invasion and metastasis,	THAP7-AS1, transcriptionally activated by SP1 and then modified by METTL3-mediated m6A, repressed the transcription of miR-22-3p and miR-320a	Oncogene	lncRNA THAP7-AS1, transcriptionally activated by SP1 and post-transcriptionally stabilized by METTL3-mediated m6A modification, exerts oncogenic properties by improving CUL4B entry into the nucleus
LINC02253	METTL3	Writer	LINC02253/METTL3/KRT18/MAPK/ERK	Growth, migration and invasion	lncRNA LINC02253 positively regulates GC growth and metastasis via increasing METTL3-mediated mRNA stability of KRT18,	Oncogene	LINC02253 activates KRT18/MAPK/ERK pathway by mediating m6A modification of KRT18 mRNA in gastric cancer
LINC01320	METTL14	Writer	METTL14/LINC01320/ miR-495-5p/RAB19	Cell viability, migration, and invasion	METTL14 regulated the m6A modification of LINC01320 and overexpressed LINC01320 regulated the miR-495-5p/RAB19 axis	Oncogene	m6A-mediated up-regulation of long noncoding RNA LINC01320 promotes the proliferation, migration, and invasion of gastric cancer via miR495-5p/RAB19 axis
lncRNA LINC00958	KIAA1429	Writer	KIAA1429/LINC00958/ GLUT1	Aerobic glycolysis	KIAA1429 catalyzed the m6A modification on LINC00958 that enhanced GLUT1 mRNA transcript stability via the m6A-dependent manner	Oncogene	m6A transferase KIAA1429-stabilized LINC00958 accelerates gastric cancer aerobic glycolysis through targeting GLUT1
lncRNA NRON	ALKBH5	Eraser	lncNRON/ALKHB5/ Nanog	Proliferation	lncNRON binds to the N6-methyladenosine eraser ALKHB5 and mediates Nanog mRNA decay	Oncogene	Long Non-Coding RNA NRON promotes Tumor Proliferation by regulating ALKBH5 and Nanog in Gastric Cancer
lncRNA NEAT1	ALKBH5	Eraser	ALKBH5/lncRNA NEAT1/EZH2	Invasion and metastasis	ALKBH5 promotes GC invasion and metastasis by demethylating the lncRNA NEAT1, influencing the expression of EZH2	Oncogene	ALKBH5 promotes invasion and metastasis of gastric cancer by decreasing methylation of the lncRNA NEAT1
lncRNA-CBSLR	YTHDF2	Reader	YTHDF2/CBSLR/YTHDF2/CBS	Ferroptosis	CBSLR interacted with YTHDF2 to form a CBSLR/YTHDF2/CBS signaling axis that decreased the stability of CBS mRNA by enhancing the binding of YTHDF2 with the m6A-modified coding sequence (CDS) of CBS mRNA		Hypoxia inducible lncRNA-CBSLR modulates ferroptosis through m6A-YTHDF2-dependent modulation of CBS in gastric cancer
microRNA-228-5p	METTL3	Writer	EED/miR-338-5p/METTL3/CDCP1	Proliferation and invasion	EED downregulated miR-338-5p through histone methylation, which in turn impaired miR-338-5p-dependent METTL3 inhibition and enhanced CDCP1 translation,	Oncogene	Methylation of microRNA-338-5p by EED promotes METTL3-mediated translation of oncogene CDCP1 in gastric cancer
miR-4429	METTL3	Writer	miR-4429/METTL3/SEC62	Proliferation and apoptosis	METTL3 interacted with SEC62 to induce the m6A on SEC62 mRNA, miR-4429 inhibited GC progression through METTL3/SEC62 axis	Suppressor	MiR-4429 prevented gastric cancer progression through targeting METTL3 to inhibit m6A-caused stabilization of SEC62
	IGF2BP1	Reader			METTL3 facilitated the stabilizing effect of IGF2BP1 on SEC62 mRNA		
miR-660			miR-660/E2F3	Proliferation and apoptosis	Overexpression of miR-660 reduced the activity of E2F3 by directly binding to E2F3 3`-UTR	Suppressor	RNA m6A modification participates in miR-660/E2F3 axis-mediated inhibition of cell proliferation in gastric cancer
circORC5	METTL14	Writer	METTL14/circORC5/miR-30c-2-3p/AKT1S1	Growth and invasion	METTL14-mediated m6A modification of circORC5 by regulating miR-30c-2-3p/AKT1S1 axis	Suppressor	METTL14-mediated m6A modification of circORC5 suppresses gastric cancer progression by regulating miR-30c-2-3p/AKT1S1 axis
circPVRL3			circPVRL3	Proliferation and migration	circPVRL3 could promote the proliferation in gastric carcinoma and have potential to encode protein	Oncogene	Down-regulation of circPVRL3 promotes the proliferation and migration of gastric cancer cells
LINC00942			LNC942/MSI2/c-MYC	Apoptosis, stemness and chemosensitivity	LNC942 stabilized c-MYC mRNA in an m6A-dependent manner. MSI2 stabilized c-MYC mRNA with m6 A modifications	Oncogene	LncRNA LINC00942 promotes chemoresistance in gastric cancer by suppressing MSI2 degradation to enhance c-MYC mRNA stability

## Biological progress of m6A modification in GC

1. Proliferation and m6A in GC

M6A RNA modification regulates growth of various tumors, including breast cancer [61], colorectal cancer [62], liver cancer [63] and gastric cancer [64]. METTL3 was significantly elevated and promoted the proliferation of gastric cancer by regulating the expression of MYC pathway in the post-transcriptional modification. METTL3 can play an oncogene role through regulating various pathways and molecules. On the one hand, by RNA-seq and m6A-seq analysis, METTL3 mediated several component molecules of MYC targeted genes which was significantly associated with several clinicopathological features and poor survival in patients with high level expression of METTL3 in GC. On the other hand, not only can METTL3 promote the proliferation as an oncogene, but also contribute to the migration and invasion of gastric cancer [40]. Consisted with the previous studies, METTL3 can enhance the MYC m6A methylation modification and increased MYC translation, which can promote the proliferation of GC [17]. Through vivo experiments and vitro experiments, EED promoted the development of GC through mediating the miR-338-5p/METTL3/CDCP1 pathway and histone methylation [65]. After knocking out METTL3 (METTL3-KO) by using CRISPR/Cas9, the expression of suppressor of cytokine signaling (SOCS) was upregulated, which promoted cell proliferation and decreased m6A RNA methylation levels [66]. Not only can METTL3 play an oncogene role in the development in tumors, but also METTL16 can promote the proliferation of GC. A recent study reports that METTL16 was upregulated in GC, exerting a crucial role in the growth of tumor in vivo, which are associated with cyclin D1 expression by blocking G1/S phase. As referred before, cyclin D1 may facilitate tumor growth as a downstream effector of METTL16 in GC [43]. KIAA1429, as the catalytic core components of the m6A methyltransferase complex, served as a scaffold and was upregulated in GC, promoting proliferation of gastric cancer in vitro and in vivo while regulating the expression of c-Jun in an m6A-independent manner [47]. miR-660, as an anti-proliferation gene to exert a regulatory role in the expression of oncogene E2F3 in GC, declines the proliferative capacity of GC cells mediated by m6A RNA modification [67]. Being different from previous findings, miR-4429 inhibited GC progression through regulating METTL3/SEC62 axis and inhibiting m6A-caused stabilization of SEC62 mRNA. Not only can m6A RNA modification participate the proliferation of GC cells on the one hand, but also affect the metastasis of GC cells on the other hand. Yang et al. [17] reported that METTL3 can potentiate the proliferation, migration and invasion of GC cells via mediating MYC mRNA m6A modification.

(2) Metastasis and m6A in GC

A growing amount of data points to the roles of m6A RNA modification in various types of cancers; EMT was frequently regarded as a molecular marker and had a significant impact on the migration of tumor cells [68]. Consisted with previous studies, METTL3 was significantly overexpressed in GC tissues in comparison to in the normal tissues. The elevated expression level of METTL3 in GC seemed to be negatively associated with E-cadherin but positively associated with N-cadherin and Vimentin, which better illustrated that METTL3 might be a candidate promotor of EMT to promote the metastasis of GC [69]. The loss of E-cadherin is commonly recognized as a molecular marker in EMT progression [70]. Song et al. preliminarily revealed the elevated HOXA10 expression mediated by METTL3 can induce EMT progression that promoted migration and invasion of GC cells in vitro and accelerated lung metastasis in vivo [71]. Stable knockdown of FTO resulted in upregulation of E-cadherin and down-regulation of vimentin in multiple GC-cell lines, which suggested that FTO may be involved in the stabilization and overexpression of EMT related genes. M6A regulators such as FTO, METTL3 may play a regulatory role in EMT progression [26]. An analysis from a microarray consisting of 90 cases revealed that the expression of FTO was significantly associated with the increase of p N stage (p = 0.001), p T stage (p = 0.001), TNM stage (p < 0.001), and liver invasion (p = 0.008). FTO-mediated m6A RNA demethylation modification participated in regulating the mitochondrial fission/fusion and metabolism to affect liver metastasis [72]. Liu et al. established the lung metastasis model and the popliteal lymph node metastasis model by injecting MGC-803 cells with METTL3 knockdown in vivo. The finding indicated that knockdown of METTL3 significantly suppressed xenograft tumor growth and lung/lymph node metastasis, which may be correlated with regulating METTL3‐PBX1‐GCH1 axis by increasing BH4 levels in GC [73]. High-expressed YTHDF1 was found in GC tissue that can promote GC progression. Based on the above evidence, Chen et al. found that GC with lung metastasis and the number of metastatic lung tumors from GC was markedly decreased After YTHDF1 knockdown [37]. Investigated by the chorioallantoic membrane (CAM) assay, METTL3-related PI3K/AKT/mTOR signaling pathway facilitated angiogenesis and carcinogenesis in GC. The finding elucidated that treatment with CM from METTL3 knockdown GC cells obviously decreased the vessel number in comparison to its control group, ADAMTS9 could be a new potential downstream target of METTL3 [74].

(3) Drug resistance and m6A in GC

A crucial finding of the current study was that ncRNAs can regulate cancer phenotypes and biological behaviors in various tumors [75]. To the best of our knowledge, symptoms in the early stages of gastric cancer are not obvious and specific so that the disease has already gone to the middle and late stages when diagnosed. Therefore, chemotherapy is occupying a dominant position in the treatment of gastric cancer. However, what makes it terrible is that tumor cell plasticity presents a huge challenge to the targeted cancer therapies, as that it continually presents enhanced chemoresistance, metastatic potential, and ability to develop tumor cell heterogeneity [76]. As for GC patients, there is no deny that tumor cell heterogeneity is playing a major part in the drug-resistance of GC therapy in recent years. A growing body of evidence indicate that m6A RNA modification plays significant roles in the drug-resistance of many different tumor cells. For instance, METTL3 can regulate the MALAT1-miR-1914-3p-YAP axis to induce drug resistance and metastasis in NSCLC [77]. Lin et al. [78] elucidated that METTL3 depletion significantly enhanced sorafenib resistance of HCC by abolishing the METTL3-mediated FOXO3 mRNA stabilization in vivo experiments. Oxaliplatin is the first-line regime for advanced gastric cancer treatment [79]. Li et al. [80] found that CD133 + CSCs are an important subset in oxaliplatin resistance of GC as that CD133 + CSCs has an ability of DNA damage repair and RNA modification via sequencing. Mechanically, CD133 + CSCs with an acquisition of oxaliplatin resistance can upregulate METTL3 to play a regulatory role in the expression stability and transcription of PARP1 at the RNA level mediated in a m6A dependent manner. Uncovered by a study, METTL3 with high expression in GC was corelated with the poor outcomes of patients and high malignancy in vitro and in vivo, which showed preferred sensitivity to everolimus and activated mTOR pathway. Everolimus, as an mTOR inhibitor, reversed the METTL3-induced proliferation in a dose-dependent manner, which may be used in the treatment of m6A/METTL3-high gastric cancer [58]. Zhang et al. [81] found that the FTO/ULK1 axis exerts significant roles in cisplatin resistance in gastric cancer. The upregulation of FTO obviously promotes cisplatin chemoresistance of gastric cancer cells (SGC-7901/DDP) whereas Knockdown of FTO significantly reverses cisplatin chemoresistance of SGC-7901/DDP cells both in vitro and in vivo, which was associated with the inhibition of Unc-51-like kinase 1 (ULK1)- mediated autophagy and the decrease of the m6 A methylated RNA level. To the best of our knowledge, a great deal evidence reveals a connection of lncRNA dysregulation with tumorigenesis, progression and chemoresistance [82]. LINC00942, as a vital chemoresistance-related lncRNA, was high expression in gastric cancer. Identified by a human proteome array and validated by RNA immunoprecipitation (RIP) and RNA pulldown assays, MSI2 has an ability that bound with lncRNA LINC00942, which exerts a regulatory role in the mechanism of cisplatin chemoresistance in GC [83]. What’ s more, FK228 can provide a strategy to overcome DDP resistance as a new MSI2 inhibitor, and the LNC942‐MSI2‐c‐MYC axis play an important role in overcoming chemoresistance in patients with GC. Omeprazole, as a king of Proton pump inhibitors (PPIs), was widely used for the treatment of peptic ulcer disease. Feng et al. reported that Omeprazole pretreatment enhanced the inhibitory effect of 5-Fu, DDP and TAX on gastric cancer cells for the first time and improved the antitumor efficiency of chemotherapeutic drugs on GC cells through the m6A-dependent mechanism that Omeprazole inhibited the expression of FTO [84]. Uncovered by these studies, m6A RNA modification exerts an extraordinary role in the chemotherapy in GC, which providing a potential strategy for addressing chemotherapy resistance in GC treatment.

(4) Aerobic glycolysis and m6A in GC

Warburg effect, also referred to as aerobic glycolysis, indicated the abnormal glucose metabolism and energy supply in tumor including gastric cancer, in which metabolic pathways are reprogrammed [85]. Yu et al. [86] performed a Glucose uptake analysis that WTAP overexpression accelerated the glucose uptake quantity in gastric cancer cells, while WTAP silencing reduced the glucose uptake quantity. Identified by MeRIP-Seq analysis and MeRIP-qPCR, HK2 is a target of WTAP that enhanced the stability of HK2 mRNA through binding with the 3’-UTR m6A site. Mediated by m6A methylation modification, LHPP regulated the metabolism of GC by changing the acetylation level featured as the Warburg effect. Many tumor cells depend on high glucose uptake rates and transport most of the glucose into lactic acid through glycolysis instead of catabolizing glucose [44].

The Warburg effect may be caused by the hypoxic microenvironment of the tumor, abnormal signaling pathways, abnormal activation of oncogenes, glucose transporters, or the overexpression of enzymes in the glycolytic pathway. LINC00958, as a classical and well-validated lncRNA driven by ectopic expression of KIAA1429, positively regulated the aerobic glycolysis of GC via recognizing the m6A modification site, which are associated with the growth of tumor in vitro assays [87]. IGF2BP3, as the m6A reader, directly recognized and bound with the m6A site of HDGF mRNA stability mediated by elevated METTL3 expression, which are correlated with tumor angiogenesis and liver metastasis by maintaining HDGF mRNA stability [41]. IGF2BP1 with high expression in stomach adenocarcinoma (STAD), enhanced the growth and metastasis of GC in vivo, which exerts an oncogenic role in GC through enhancing the glycolytic enzyme c-Myc. Glucose uptake and lactate production analysis revealed that IGF2BP1 overexpression promoted the glucose uptake and lactate production quantitation, while IGF2BP1 knockdown repressed the glucose and lactate [39]. As mentioned above that m6A regulators play a regulatory role in the aerobic glycolysis of GC.

(5) TME and m6A in GC

An immunity-related analysis demonstrated that lncRNAs m6Amodification might negatively modulate the expression of immune checkpoint (PD-1 and CTLA4) [88]. Loss of YTHDF1 in GC tumors leads to recruitment of mature dendritic cells (DCs) with the increased expression of MHC II and the secretion of interleukin-12 (IL-12), which promoted CD4^+^ and CD8^+^ T cells infiltration as increased interferon-γ (IFN-γ) secretion in turn. Loss of YTHDF1 trigger in the host’s antitumor immunity [89]. High expression of WTAP is correlated with a low T cell–related immune response, which indicated a poor prognosis and exerted a regulatory in RNA methylation [18]. Highly consistent with the three immune phenotypes of tumors including immune-excluded, immune-inflamed and immune-desert phenotypes, the TME cell-infiltrating characteristics under these three patterns were various, which can predict stages of tumor inflammation, subtypes, TME stromal activity, genetic variation, and patient prognosis [90]. Song et al. [91] collected a total of 933 GC samples from the Gene Expression Omnibus (GEO) and TCGA cohort and established a complete scoring system that are used to analyze the relationship between the prognosis of GC and tumor microenvironment (TME), reaching a conclusion that RNA adenosine modifications strongly linked with TME cell infiltration, survival advantage, CD4^−^ T-cell infiltration, high tumor mutation burden, and cell cycle signaling pathways. What’s more, Wang et al. [55] established an m6A-LPS prognostic model containing 14 m6A-related lncRNA pairs, which showed high and independent clinical prediction value in GC patients. The immune infiltration analysis revealed that resting memory CD4 T cells, resting dendritic cells, monocytes, and M2 macrophages were positively related to the risk score. Not only m6A RNA modification can affect the tumor microenvironment by regulating downstream targeted molecules, but also influence lincRNAs, which playing a crucial role in regulating tumor microenvironment (TME) infiltration and are benefit for immunotherapy and chemotherapy [92]. Using the LASSO algorithm, high-risk patients are associated with more severe infiltration of cancer-associated fibroblasts, endothelial cells, macrophages, particularly M2 macrophages, and monocytes in comparation to in low-risk patients [93].

## Application of m6A modification in GC

m6A-related lncRNAs exert a significant role in the development of various tumors, such as gastric cancer, NSCLC and osteosarcoma. Huang et al. established a prognostic signature comprising 14 m6A-related lncRNAs in STAD. From the prognostic signature, it can be clearly evaluated that the prognosis and role of m6A-related lncRNAs in STAD. Not only can it predict the OS of patients, clinicopathological characteristics, TME, ICGs expression, and the response to ICIs on the one hand, but also identify the biological processes and pathways associated with m6A-related lncRNAs in STAD on the other hand [94]. Similarly, Zhang et al. [95] also constructed a diagnostic signature m6A-miRNAs used in the detection of cancer with high accuracy, which exerted a crucial role in the large-scale cancer screening. Level of m6A in serum is a great diagnostic biomarker for GC patients. Based on two m6A-related lncRNAs in a recent study, the research established a risk model used in the prediction of survival of patients with OS, which can target downstream molecules in the pathogenesis of OS [96]. There is a connection in m6A-related lncRNAs and immune infiltration, immunotherapy, and chemotherapy in GC. Risk score compared with other clinical factors. (eg, age, sex, grade, and stage) was used to predict the prognosis of GC patients, while high expression of programmed death-1 (PD-1) and cytotoxic T-lymphocyte associated protein 4 (CTLA4) showed low risk scores [92]. A joint analysis revealed that the relation between lncRNA m6A methylome and lncRNA/mRNA expression profiles in GC, which better provided diagnosis to the GC patients [97]. With the development of high-throughput sequencing and bioinformatics in tumor study, research can assess the prognosis of patients in GC by using machine learning algorithm such as the LASSO algorithm. Based on a 11-m6A-related lncRNA signature, a new risk model can predict the clinicopathological features, tumor-infiltrating immune cells, and biomarkers of immune checkpoint inhibitors in different risk groups [93]. m6A-related lncRNAs had superior values in optimizing ICIs therapy which is used to predict patients’ outcomes of ICI therapy and the prognosis of gastric cancer. Interestingly, m6A-related lncRNAs offered new insights to the progression of immunotherapy in GC [98]. m6A-related lncRNA signature can be used as an independent prognostic marker for GC [99].

## Conclusions

In summary, with the increasing studies of transcriptomics in tumors, m6ARNA modification exerts an crucial role in the occurrence, progression and development of tumors, which better elucidating the mechanism of tumorigenesis. m6A modification, as the most significant post-transcriptional modification, played a pro-tumorigenic as well as an anti-tumorigenic role in the pathogenic mechanism of GC. To best of our knowledge, m6A RNA modification regulators such as Writers, Erasers and Readers all participated in the regulation of GC in an m6A dependent manner. Not only mRNA but also ncRNAs all participated in the regulation of m6A regulators as downstream molecules or targets in GC. Aberrant expression of ncRNAs mediated by the expression of m6Aregulators in GC will affect the methylation level of downstream targeted RNAs, which exerted a regulatory role in the proliferation, apoptosis, migration, invasion and metastasis of GC. What’s more important, GC-related molecules also regulate the biological progression such as EMT, aerobic glycolysis, drug resistance and Tumor microenvironment constitution in GC. M6A regulators was applied in the diagnose, therapy and Survival prediction of GC. As mentioned above, the increase in total RNA m6A levels in GC is a complex result of various expression of writers, readers, and erasers. It is not sufficient to clearly explain the pathogenic mechanism of m6A RNA modification in gastric cancer by merely exploring m6A regulators. However, Future research should concentrate on the novel molecular therapies targeting the pathways of m6A modification, which will provide better comprehension for GC mechanism.

## Data Availability

The dataset supporting the conclusion of this article is included within the article.

## Abbreviations

GC: Gastric cancer
PDGA: Poorly differentiated adenocarcinoma of the stomach
GCTs: Gastric cancer tissues
ANTs: Adjacent normal tissues
AIMTs: Adjacent intestinal metaplasia tissues
m6A: N6-Methyladenosine
m5C: 5-Methylcytidine
m1A: N1-methyladenosine
m6Am: N6, 2′-o-dimethyladenosine
TME: Tumor microenvironment
EMT: Epithelial-mesenchymal transition
TCGA: The cancer genome atlas
GEO: Gene expression omnibus
GO: Gene ontology
GSEA: Gene set enrichment analysis
SNP: Single-nucleotide polymorphism
LASSO: Least absolute shrinkage and selection operator
ncRNA: Non-coding RNA
lncRNA: Long non-coding RNA
lincRNA: Long intergenic non-coding RNA
miRNAs: MicroRNAs
circRNAs: Circular RNAs
rRNAs: Ribosomal RNAs
mRNA: Messenger RNA
tRNA: Transfer RNA
snRNA: Small nuclear RNAs
3’ UTRs: 3’ Untranslated regions
MeRIP-Seq: Methylated RNA immunoprecipitation next generation sequencing
MeRIP-seq: Methylated RNA immunoprecipitation sequencing
LAIC-seq: M6A-level and isoform-characterization sequencing
miCLIP-seq: M6A individual-nucleotide-resolution cross-linking and immunoprecipitation sequencing
LC–MS/MS: Liquid chromatography-tandem mass spectrometry
RIP: RNA immunoprecipitation
IHC: Immunohistochemistry
Seq: Sequence
APA: Alternative polyadenylation
METTL3: Methyltransferase like 3
SAM: S-adenosyl methionine
METTL14: Methyltransferase like 14
WTAP: Wilms tumor 1-associated protein
METTL16: Methyltransferase like 16
MALAT1: Metastasis-associated lung adenocarcinoma transcript 1
YTH: YT521-B homology
IGF2BPs: Insulin-like growth factor 2 mRNA-binding proteins
eIF3: Eukaryotic Initiation factor 3
FTO: Fat mass and obesity-associated protein
ALKBH5: α-Ketoglutarate-dependent dioxygenase homolog 5
HNRNPs: Heterogeneous nuclear ribonucleoproteins
ZC3H13: Zinc finger CCCH domain-containing protein 13
MYC: Myelocytomatosis viral oncogene homolog
HDAC3: Histone deacetylase 3
FOXA2: Forkhead box transcription factor A2
ZMYM1: Zinc finger MYM-type containing 1
HDGF: Hepatoma-derived growth factor
BATF2: Basic leucine zipper ATF-like transcription factor 2
FZD7: Frizzled 7
E2F3: E2F transcription factor 3
EED: Embryonic ectoderm development protein
PBX1: Pre‐B‐cell leukemia homeobox 1
ZNF668: Zinc finger protein 668
GCH1: GTP cyclohydrolase 1
MHC: Major histocompatibility complex
OS: Overall survival
PFS: Progression free survival

## Code availability

Not application.

## References

[1] Sung H, Ferlay J, Siegel RL, Laversanne M, Soerjomataram I, Jemal A, Bray F (2021). Global Cancer Statistics 2020: GLOBOCAN estimates of incidence and mortality worldwide for 36 cancers in 185 countries. CA Cancer J Clin.

[2] Lan Q, Liu PY, Haase J, Bell JL, Hüttelmaier S, Liu T (2019). The critical role of RNA m(6)A methylation in cancer. Can Res.

[3] Wei CM, Gershowitz A, Moss B (1975). Methylated nucleotides block 5’ terminus of HeLa cell messenger RNA. Cell.

[4] Lee Y, Choe J, Park OH, Kim YK (2020). Molecular mechanisms driving mRNA degradation by m(6)A modification. Trends Genet.

[5] Zhao BS, Roundtree IA, He C (2017). Post-transcriptional gene regulation by mRNA modifications. Nat Rev Mol Cell Biol.

[6] Sikorski V, Selberg S, Lalowski M, Karelson M, Kankuri E (2023). The structure and function of YTHDF epitranscriptomic m(6)A readers. Trends Pharmacol Sci.

[7] Bokar JA, Shambaugh ME, Polayes D, Matera AG, Rottman FM (1997). Purification and cDNA cloning of the AdoMet-binding subunit of the human mRNA (N6-adenosine)-methyltransferase. RNA.

[8] Oerum S, Meynier V, Catala M, Tisné C (2021). A comprehensive review of m6A/m6Am RNA methyltransferase structures. Nucleic Acids Res.

[9] Pendleton KE, Chen B, Liu K, Hunter OV, Xie Y, Tu BP, Conrad NK (2017). The U6 snRNA m(6)A methyltransferase METTL16 regulates SAM synthetase intron retention. Cell.

[10] Jia G, Fu Y, Zhao X, Dai Q, Zheng G, Yang Y, Yi C, Lindahl T, Pan T, Yang YG (2011). N6-methyladenosine in nuclear RNA is a major substrate of the obesity-associated FTO. Nat Chem Biol.

[11] Aik W, Scotti JS, Choi H, Gong L, Demetriades M, Schofield CJ, McDonough MA (2014). Structure of human RNA N^6^-methyladenine demethylase ALKBH5 provides insights into its mechanisms of nucleic acid recognition and demethylation. Nucleic Acids Res.

[12] Zou S, Toh JD, Wong KH, Gao YG, Hong W, Woon EC (2016). N(6)-Methyladenosine: a conformational marker that regulates the substrate specificity of human demethylases FTO and ALKBH5. Sci Rep.

[13] Wu R, Liu Y, Yao Y, Zhao Y, Bi Z, Jiang Q, Liu Q, Cai M, Wang F, Wang Y (2018). FTO regulates adipogenesis by controlling cell cycle progression via m(6)A-YTHDF2 dependent mechanism. Biochim Biophys Acta Mol Cell Biol Lipids.

[14] Lin S, Liu J, Jiang W, Wang P, Sun C, Wang X, Chen Y, Wang H (2019). METTL3 promotes the proliferation and mobility of gastric cancer cells. Open Med.

[15] Zhang HM, Qi FF, Wang J, Duan YY, Zhao LL, Wang YD, Zhang TC, Liao XH (2022). The m6A methyltransferase METTL3-mediated N6-methyladenosine modification of DEK mRNA to promote gastric cancer cell growth and metastasis. Int J Mol Sci.

[16] Liu T, Yang S, Sui J, Xu SY, Cheng YP, Shen B, Zhang Y, Zhang XM, Yin LH, Pu YP (2020). Dysregulated N6-methyladenosine methylation writer METTL3 contributes to the proliferation and migration of gastric cancer. J Cell Physiol.

[17] Yang Z, Jiang X, Li D, Jiang X (2020). HBXIP promotes gastric cancer via METTL3-mediated MYC mRNA m6A modification. Aging.

[18] Li H, Su Q, Li B, Lan L, Wang C, Li W, Wang G, Chen W, He Y, Zhang C (2020). High expression of WTAP leads to poor prognosis of gastric cancer by influencing tumour-associated T lymphocyte infiltration. J Cell Mol Med.

[19] Su Y, Huang J, Hu J (2019). m(6)A RNA methylation regulators contribute to malignant progression and have clinical prognostic impact in gastric cancer. Front Oncol.

[20] Zhang C, Zhang M, Ge S, Huang W, Lin X, Gao J, Gong J, Shen L (2019). Reduced m6A modification predicts malignant phenotypes and augmented Wnt/PI3K-Akt signaling in gastric cancer. Cancer Med.

[21] Liu X, Xiao M, Zhang L, Li L, Zhu G, Shen E, Lv M, Lu X, Sun Z (2021). The m6A methyltransferase METTL14 inhibits the proliferation, migration, and invasion of gastric cancer by regulating the PI3K/AKT/mTOR signaling pathway. J Clin Lab Anal.

[22] Fan HN, Chen ZY, Chen XY, Chen M, Yi YC, Zhu JS, Zhang J (2022). METTL14-mediated m(6)A modification of circORC5 suppresses gastric cancer progression by regulating miR-30c-2-3p/AKT1S1 axis. Mol Cancer.

[23] Wang Z, Liu J, Yang Y, Xing C, Jing J, Yuan Y (2021). Expression and prognostic potential of ribosome 18S RNA m(6)A methyltransferase METTL5 in gastric cancer. Cancer Cell Int.

[24] Xu X, Zhou E, Zheng J, Zhang C, Zou Y, Lin J, Yu J (2021). Prognostic and predictive value of m6A “Eraser” related gene signature in gastric cancer. Front Oncol.

[25] Ge L, Zhang N, Chen Z, Song J, Wu Y, Li Z, Chen F, Wu J, Li D, Li J (2020). Level of N6-methyladenosine in peripheral blood RNA: a novel predictive biomarker for gastric cancer. Clin Chem.

[26] Shimura T, Kandimalla R, Okugawa Y, Ohi M, Toiyama Y, He C, Goel A (2022). Novel evidence for m(6)A methylation regulators as prognostic biomarkers and FTO as a potential therapeutic target in gastric cancer. Br J Cancer.

[27] Li Y, Zhou D, Liu Q, Zhu W, Ye Z, He C (2022). Gene polymorphisms of m6A erasers FTO and ALKBH1 associated with susceptibility to gastric cancer. Pharmacogenom Personal Med.

[28] Li Y, Zheng D, Wang F, Xu Y, Yu H, Zhang H (2019). Expression of demethylase genes, FTO and ALKBH1, is associated with prognosis of gastric cancer. Dig Dis Sci.

[29] Hu Y, Gong C, Li Z, Liu J, Chen Y, Huang Y, Luo Q, Wang S, Hou Y, Yang S (2022). Demethylase ALKBH5 suppresses invasion of gastric cancer via PKMYT1 m6A modification. Mol Cancer.

[30] Wang S, Wang Y, Zhang Z, Zhu C, Wang C, Yu F, Zhao E (2021). Long non-coding RNA NRON promotes tumor proliferation by regulating ALKBH5 and nanog in gastric cancer. J Cancer.

[31] Yue B, Cui R, Zheng R, Jin W, Song C, Bao T, Wang M, Yu F, Zhao E (2021). Essential role of ALKBH5-mediated RNA demethylation modification in bile acid-induced gastric intestinal metaplasia. Mol Ther Nucleic Acids.

[32] Guan K, Liu X, Li J, Ding Y, Li J, Cui G, Cui X, Sun R (2020). Expression status and prognostic value Of M6A-associated genes in gastric cancer. J Cancer.

[33] Pi J, Wang W, Ji M, Wang X, Wei X, Jin J, Liu T, Qiang J, Qi Z, Li F (2021). YTHDF1 promotes gastric carcinogenesis by controlling translation of FZD7. Can Res.

[34] Shen X, Zhao K, Xu L, Cheng G, Zhu J, Gan L, Wu Y, Zhuang Z (2020). YTHDF2 inhibits gastric cancer cell growth by regulating FOXC2 signaling pathway. Front Genet.

[35] Liu T, Yang S, Cheng YP, Kong XL, Du DD, Wang X, Bai YF, Yin LH, Pu YP, Liang GY (2020). The N6-methyladenosine (m6A) methylation gene YTHDF1 reveals a potential diagnostic role for gastric cancer. Cancer Manag Res.

[36] Sang L, Sun L, Wang A, Zhang H, Yuan Y (2020). The N6-methyladenosine features of mRNA and aberrant expression of m6A modified genes in gastric cancer and their potential impact on the risk and prognosis. Front Genet.

[37] Chen XY, Liang R, Yi YC, Fan HN, Chen M, Zhang J, Zhu JS (2021). The m(6)A reader YTHDF1 facilitates the tumorigenesis and metastasis of gastric cancer via USP14 translation in an m(6)A-dependent manner. Front Cell Dev Biol.

[38] de Souza CR, Leal MF, Calcagno DQ, Costa Sozinho EK, Borges Bdo N, Montenegro RC, Dos Santos AK, Dos Santos SE, Ribeiro HF, Assumpção PP (2013). MYC deregulation in gastric cancer and its clinicopathological implications. PLoS ONE.

[39] Luo F, Lin K (2022). N(6)-methyladenosine (m(6)A) reader IGF2BP1 accelerates gastric cancer aerobic glycolysis in c-Myc-dependent manner. Exp Cell Res.

[40] Yang DD, Chen ZH, Yu K, Lu JH, Wu QN, Wang Y, Ju HQ, Xu RH, Liu ZX, Zeng ZL (2020). METTL3 promotes the progression of gastric cancer via targeting the MYC pathway. Front Oncol.

[41] Wang Q, Chen C, Ding Q, Zhao Y, Wang Z, Chen J, Jiang Z, Zhang Y, Xu G, Zhang J (2020). METTL3-mediated m(6)A modification of HDGF mRNA promotes gastric cancer progression and has prognostic significance. Gut.

[42] Zhou W, Xian Q, Wang Q, Wu C, Yan H, Li X, Lu L, Wu C, Zhu D, Xu X (2021). m6A methyltransferase 3 promotes the proliferation and migration of gastric cancer cells through the m6A modification of YAP1. J Oncol.

[43] Wang XK, Zhang YW, Wang CM, Li B, Zhang TZ, Zhou WJ, Cheng LJ, Huo MY, Zhang CH, He YL (2021). METTL16 promotes cell proliferation by up-regulating cyclin D1 expression in gastric cancer. J Cell Mol Med.

[44] Lin JX, Lian NZ, Gao YX, Zheng QL, Yang YH, Ma YB, Xiu ZS, Qiu QZ, Wang HG, Zheng CH (2022). m6A methylation mediates LHPP acetylation as a tumour aerobic glycolysis suppressor to improve the prognosis of gastric cancer. Cell Death Dis.

[45] Xia TL, Li X, Wang X, Zhu YJ, Zhang H, Cheng W, Chen ML, Ye Y, Li Y, Zhang A (2021). N(6)-methyladenosine-binding protein YTHDF1 suppresses EBV replication and promotes EBV RNA decay. EMBO Rep.

[46] Wang D, Qu X, Lu W, Wang Y, Jin Y, Hou K, Yang B, Li C, Qi J, Xiao J (2021). N(6)-Methyladenosine RNA demethylase FTO promotes gastric cancer metastasis by down-regulating the m6a methylation of ITGB1. Front Oncol.

[47] Miao R, Dai CC, Mei L, Xu J, Sun SW, Xing YL, Wu LS, Wang MH, Wei JF (2020). KIAA1429 regulates cell proliferation by targeting c-Jun messenger RNA directly in gastric cancer. J Cell Physiol.

[48] Yuan W, Chen S, Li B, Han X, Meng B, Zou Y, Chang S (2022). The N6-methyladenosine reader protein YTHDC2 promotes gastric cancer progression via enhancing YAP mRNA translation. Transl Oncol.

[49] Wang X, Li G, Luo Q, Xie J, Gan C (2018). Integrated TCGA analysis implicates lncRNA CTB-193M12.5 as a prognostic factor in lung adenocarcinoma. Cancer Cell Int.

[50] Yang G, Lu X, Yuan L (2014). LncRNA: a link between RNA and cancer. Biochem Biophys Acta.

[51] He C (2010). Grand challenge commentary: RNA epigenetics?. Nat Chem Biol.

[52] Liu HT, Zou YX, Zhu WJ, Sen L, Zhang GH, Ma RR, Guo XY, Gao P (2022). lncRNA THAP7-AS1, transcriptionally activated by SP1 and post-transcriptionally stabilized by METTL3-mediated m6A modification, exerts oncogenic properties by improving CUL4B entry into the nucleus. Cell Death Differ.

[53] Gao Z, Long Y, Wu Y, Pu Y, Xue F (2022). LncRNA LINC02253 activates KRT18/MAPK/ERK pathway by mediating N6-methyladenosine modification of KRT18 mRNA in gastric cancer. Carcinogenesis.

[54] Zhang J, Guo S, Piao HY, Wang Y, Wu Y, Meng XY, Yang D, Zheng ZC, Zhao Y (2019). ALKBH5 promotes invasion and metastasis of gastric cancer by decreasing methylation of the lncRNA NEAT1. J Physiol Biochem.

[55] Wang JM, Li X, Yang P, Geng WB, Wang XY (2022). Identification of a novel m6A-related lncRNA pair signature for predicting the prognosis of gastric cancer patients. BMC Gastroenterol.

[56] Hu N, Ji H (2021). N6-methyladenosine (m6A)-mediated up-regulation of long noncoding RNA LINC01320 promotes the proliferation, migration, and invasion of gastric cancer via miR495-5p/RAB19 axis. Bioengineered.

[57] He H, Wu W, Sun Z, Chai L (2019). MiR-4429 prevented gastric cancer progression through targeting METTL3 to inhibit m(6)A-caused stabilization of SEC62. Biochem Biophys Res Commun.

[58] Sun Y, Li S, Yu W, Zhao Z, Gao J, Chen C, Wei M, Liu T, Li L, Liu L (2020). N(6)-methyladenosine-dependent pri-miR-17-92 maturation suppresses PTEN/TMEM127 and promotes sensitivity to everolimus in gastric cancer. Cell Death Dis.

[59] Sun HD, Xu ZP, Sun ZQ, Zhu B, Wang Q, Zhou J, Jin H, Zhao A, Tang WW, Cao XF (2018). Down-regulation of circPVRL3 promotes the proliferation and migration of gastric cancer cells. Sci Rep.

[60] Zhang C, Wang J, Geng X, Tu J, Gao H, Li L, Zhou X, Wu H, Jing J, Pan W (2020). Circular RNA expression profile and m6A modification analysis in poorly differentiated adenocarcinoma of the stomach. Epigenomics.

[61] Cai X, Wang X, Cao C, Gao Y, Zhang S, Yang Z, Liu Y, Zhang X, Zhang W, Ye L (2018). HBXIP-elevated methyltransferase METTL3 promotes the progression of breast cancer via inhibiting tumor suppressor let-7g. Cancer Lett.

[62] Yao B, Zhang Q, Yang Z, An F, Nie H, Wang H, Yang C, Sun J, Chen K, Zhou J (2022). CircEZH2/miR-133b/IGF2BP2 aggravates colorectal cancer progression via enhancing the stability of m(6)A-modified CREB1 mRNA. Mol Cancer.

[63] Zhang C, Huang S, Zhuang H, Ruan S, Zhou Z, Huang K, Ji F, Ma Z, Hou B, He X (2020). YTHDF2 promotes the liver cancer stem cell phenotype and cancer metastasis by regulating OCT4 expression via m6A RNA methylation. Oncogene.

[64] Zhang N, Zuo Y, Peng Y, Zuo L (2021). Function of N6-methyladenosine modification in tumors. J Oncol.

[65] Zhang F, Yan Y, Cao X, Zhang J, Li Y, Guo C (2021). Methylation of microRNA-338-5p by EED promotes METTL3-mediated translation of oncogene CDCP1 in gastric cancer. Aging.

[66] Jiang L, Chen T, Xiong L, Xu JH, Gong AY, Dai B, Wu G, Zhu K, Lu E, Mathy NW (2020). Knockdown of m6A methyltransferase METTL3 in gastric cancer cells results in suppression of cell proliferation. Oncol Lett.

[67] He X, Shu Y (2019). RNA N6-methyladenosine modification participates in miR-660/E2F3 axis-mediated inhibition of cell proliferation in gastric cancer. Pathol Res Pract.

[68] Thiery JP, Acloque H, Huang RY, Nieto MA (2009). Epithelial-mesenchymal transitions in development and disease. Cell.

[69] Yue B, Song C, Yang L, Cui R, Cheng X, Zhang Z, Zhao G (2019). METTL3-mediated N6-methyladenosine modification is critical for epithelial-mesenchymal transition and metastasis of gastric cancer. Mol Cancer.

[70] Bure IV, Nemtsova MV, Zaletaev DV (2019). Roles of E-cadherin and noncoding RNAs in the epithelial-mesenchymal transition and progression in gastric cancer. Int J Mol Sci.

[71] Song C, Zhou C (2021). HOXA10 mediates epithelial-mesenchymal transition to promote gastric cancer metastasis partly via modulation of TGFB2/Smad/METTL3 signaling axis. J Exp Clin Cancer Res.

[72] Zhou Y, Wang Q, Deng H, Xu B, Zhou Y, Liu J, Liu Y, Shi Y, Zheng X, Jiang J (2022). N6-methyladenosine demethylase FTO promotes growth and metastasis of gastric cancer via m(6)A modification of caveolin-1 and metabolic regulation of mitochondrial dynamics. Cell Death Dis.

[73] Liu Y, Zhai E, Chen J, Qian Y, Zhao R, Ma Y, Liu J, Huang Z, Cai S, Chen J (2022). m(6) A-mediated regulation of PBX1-GCH1 axis promotes gastric cancer proliferation and metastasis by elevating tetrahydrobiopterin levels. Cancer Commun.

[74] Wang N, Huo X, Zhang B, Chen X, Zhao S, Shi X, Xu H, Wei X (2022). METTL3-mediated ADAMTS9 suppression facilitates angiogenesis and carcinogenesis in gastric cancer. Front Oncol.

[75] Ma S, Chen C, Ji X, Liu J, Zhou Q, Wang G, Yuan W, Kan Q, Sun Z (2019). The interplay between m6A RNA methylation and noncoding RNA in cancer. J Hematol Oncol.

[76] Plaks V, Kong N, Werb Z (2015). The cancer stem cell niche: how essential is the niche in regulating stemness of tumor cells?. Cell Stem Cell.

[77] Jin D, Guo J, Wu Y, Du J, Yang L, Wang X, Di W, Hu B, An J, Kong L (2021). m(6)A mRNA methylation initiated by METTL3 directly promotes YAP translation and increases YAP activity by regulating the MALAT1-miR-1914-3p-YAP axis to induce NSCLC drug resistance and metastasis. J Hematol Oncol.

[78] Lin Z, Niu Y, Wan A, Chen D, Liang H, Chen X, Sun L, Zhan S, Chen L, Cheng C (2020). RNA m(6) A methylation regulates sorafenib resistance in liver cancer through FOXO3-mediated autophagy. EMBO J.

[79] Al-Batran SE, Homann N, Pauligk C, Goetze TO, Meiler J, Kasper S, Kopp HG, Mayer F, Haag GM, Luley K (2019). Perioperative chemotherapy with fluorouracil plus leucovorin, oxaliplatin, and docetaxel versus fluorouracil or capecitabine plus cisplatin and epirubicin for locally advanced, resectable gastric or gastro-oesophageal junction adenocarcinoma (FLOT4): a randomised, phase 2/3 trial. Lancet.

[80] Li H, Wang C, Lan L, Yan L, Li W, Evans I, Ruiz EJ, Su Q, Zhao G, Wu W (2022). METTL3 promotes oxaliplatin resistance of gastric cancer CD133+ stem cells by promoting PARP1 mRNA stability. Cell Mol Life Sci.

[81] Zhang Y, Gao LX, Wang W, Zhang T, Dong FY, Ding WP (2022). M(6) A demethylase fat mass and obesity-associated protein regulates cisplatin resistance of gastric cancer by modulating autophagy activation through ULK1. Cancer Sci.

[82] Liu K, Gao L, Ma X, Huang JJ, Chen J, Zeng L, Ashby CR, Zou C, Chen ZS (2020). Long non-coding RNAs regulate drug resistance in cancer. Mol Cancer.

[83] Zhu Y, Zhou B, Hu X, Ying S, Zhou Q, Xu W, Feng L, Hou T, Wang X, Zhu L (2022). LncRNA LINC00942 promotes chemoresistance in gastric cancer by suppressing MSI2 degradation to enhance c-Myc mRNA stability. Clin Transl Med.

[84] Feng S, Qiu G, Yang L, Feng L, Fan X, Ren F, Huang K, Chen Y (2021). Biosci Rep.

[85] Huang S, Guo Y, Li Z, Zhang Y, Zhou T, You W, Pan K, Li W (2020). A systematic review of metabolomic profiling of gastric cancer and esophageal cancer. Cancer Biol Med.

[86] Yu H, Zhao K, Zeng H, Li Z, Chen K, Zhang Z, Li E, Wu Z (2021). N(6)-methyladenosine (m(6)A) methyltransferase WTAP accelerates the Warburg effect of gastric cancer through regulating HK2 stability. Biomed Pharmacother.

[87] Yang D, Chang S, Li F, Ma M, Yang J, Lv X, Huangfu L, Jia C (2021). m(6) A transferase KIAA1429-stabilized LINC00958 accelerates gastric cancer aerobic glycolysis through targeting GLUT1. IUBMB Life.

[88] Han T, Xu D, Zhu J, Li J, Liu L, Deng Y (2021). Identification of a robust signature for clinical outcomes and immunotherapy response in gastric cancer: based on N6-methyladenosine related long noncoding RNAs. Cancer Cell Int.

[89] Bai X, Wong CC, Pan Y, Chen H, Liu W, Zhai J, Kang W, Shi Y, Yamamoto M, Tsukamoto T (2022). Loss of YTHDF1 in gastric tumors restores sensitivity to antitumor immunity by recruiting mature dendritic cells. J Immunother Cancer.

[90] Zhang B, Wu Q, Li B, Wang D, Wang L, Zhou YL (2020). m(6)A regulator-mediated methylation modification patterns and tumor microenvironment infiltration characterization in gastric cancer. Mol Cancer.

[91] Song P, Zhou S, Qi X, Jiao Y, Gong Y, Zhao J, Yang H, Qian Z, Qian J, Tang L (2022). RNA modification writers influence tumor microenvironment in gastric cancer and prospects of targeted drug therapy. J Bioinform Comput Biol.

[92] Huang J, Chen W, Chen C, Jie Z, Xiao T (2022). Comprehensive analysis and prognosis prediction of N6-methyladenosine-related lncRNAs in immune microenvironment infiltration of gastric cancer. Int J Gen Med.

[93] Lei L, Li N, Yuan P, Liu D (2022). A new risk model based on a 11-m(6)A-related lncRNA signature for predicting prognosis and monitoring immunotherapy for gastric cancer. BMC Cancer.

[94] Huang Y, Yang Z, Huang C, Jiang X, Yan Y, Zhuang K, Wen Y, Liu F, Li P (2021). Identification of N6-methylandenosine-related lncRNAs for subtype identification and risk stratification in gastric adenocarcinoma. Front Oncol.

[95] Zhang B, Chen Z, Tao B, Yi C, Lin Z, Li Y, Shao W, Lin J, Chen J (2021). m(6)A target microRNAs in serum for cancer detection. Mol Cancer.

[96] Yang K, Wang F, Li K, Peng G, Yang H, Xu H, Xiang Y, Sun H (2022). N6-methyladenosine modification-related long non-coding RNAs are potential biomarkers for predicting the prognosis of patients with osteosarcoma. Technol Cancer Res Treat.

[97] Lv Z, Sun L, Xu Q, Xing C, Yuan Y (2020). Joint analysis of lncRNA m(6)A methylome and lncRNA/mRNA expression profiles in gastric cancer. Cancer Cell Int.

[98] Wang Y, Zhu GQ, Tian D, Zhou CW, Li N, Feng Y, Zeng MS (2022). Comprehensive analysis of tumor immune microenvironment and prognosis of m6A-related lncRNAs in gastric cancer. BMC Cancer.

[99] Wang H, Meng Q, Ma B (2021). Characterization of the prognostic m6A-related lncRNA signature in gastric cancer. Front Oncol.

